# Is synovial calprotectin lateral flow test a reliable intraoperative biomarker for periprosthetic joint infection? A systematic review and meta-analysis

**DOI:** 10.1007/s00402-026-06202-w

**Published:** 2026-02-10

**Authors:** Konstantinos Giatroudakis, Efthymios Iliopoulos, Konstantinos Kateros, Athanasios Ververidis

**Affiliations:** 1 1st Orthopaedic Surgery Department, Athens General Hospital “G. Gennimatas”, Athens, Greece; 2 University Orthopaedic Surgery Department, Faculty of Medicine, Democritus University of Thrace, University General Hospital of Alexandroupolis, Alexandroupolis, Greece

**Keywords:** Synovial calprotectin, Joint arthroplasty, Periprosthetic joint infection, Biomarkers

## Abstract

**Introduction:**

Periprosthetic Joint Infection (PJI) remains one of the most challenging complications in arthroplasty, often leading to diagnostic uncertainty and suboptimal treatment decisions. Synovial calprotectin has emerged as a promising biomarker, with the lateral flow test (LFT) offering real-time results for intraoperative decision-making. This meta-analysis aimed to evaluate the diagnostic accuracy of intraoperative synovial calprotectin LFT in detecting PJI.

**Materials and methods:**

A systematic review and meta-analysis were conducted according to PRISMA guidelines. Thirteen studies comprising 990 patients were included. Pooled sensitivity, specificity, positive and negative predictive values (PPV, NPV), likelihood ratios (LR+, LR−), diagnostic odds ratio (DOR), and area under the curve (AUC) were calculated using a random-effects model. Subgroup analyses were performed based on test kit type and calprotectin cutoff values.

**Results:**

The pooled sensitivity and specificity of synovial calprotectin LFT were 88% (95% CI: 82–92%) and 89% (95% CI: 83–93%), respectively, with an AUC of 0.94. The DOR was 56.1 (95% CI: 28.4-110.8). The ≥ 50 mg/L cutoff subgroup demonstrated superior diagnostic performance across all metrics. Despite subgroup analysis, heterogeneity remained high (I² = 74.1%), likely due to differences in reference standards, joint types, and study design.

**Conclusions:**

Intraoperative synovial calprotectin LFT is a highly accurate and rapid diagnostic tool for PJI, with strong applicability in real-world surgical settings. Its cost-effectiveness and ease of use support its integration as a valuable adjunctive marker within standard diagnostic algorithms, providing supplementary evidence when conventional criteria remain inconclusive.

## Introduction

Periprosthetic Joint Infection (PJI) constitutes a potentially disastrous complication of Total Hip and Knee Replacement (THR, TKR). With an incidence of 1–3% and a continuous increase in arthroplasty procedures, the financial burden on the United States healthcare system is substantial with projected annual costs expected to reach $1.85 billion by 2030, posing a substantial financial burden [1, 2]. Despite evolutionary changes in the diagnostic algorithms, definition criteria, and methods of infection detection, PJI remains a challenging diagnostic entity, often leading to extended antibiotic administration and wrong surgical decisions. Consequently, patients who are mistakenly assumed to have a PJI undergo needless surgical procedures and antibiotic treatments, while those who are mistakenly assumed to be aseptic suffer from persistent infections and frequently unsuccessful operations. In turn, the limitations of widely used algorithms often lead to increased use of costly imaging and tests to exclude infection, which exhibit the same deficiencies as the established criteria [3]. Thus, the PJI-associated mortality rate is of great concern, with the one-year postoperative mortality rate after a two-stage revision being 4.22% and gradually increasing to reach a 21.12% mortality rate at the five-year milestone [4].

The challenge in detecting a PJI has been extensively recognized in the literature. Widely accepted criteria [i. of Musculoskeletal Infection Society (MSIS) criteria in 2011 and 2018, ii. of International Consensus Meeting on Periprosthetic Joint Infection (ICM) criteria in 2013 and 2018, iii. of Infectious Diseases Society of America (IDSM) in 2013, iv. of European Bone and Joint Infection Society (EBJIS) in 2021] have been proposed for the diagnosis of PJI, with the majority of these definitions relying on the same set of diagnostic findings [5–9]. A combination of clinical, laboratory, and radiologic findings has to be met for the diagnosis of PJI. Although, in the case of sinus tract communication, the diagnosis may easily be confirmed, in most cases pathologic findings have to be investigated from serum and synovial fluid traditional biomarkers [i.e. Erythrocyte Sedimentation Rate (ESR), C-Reactive Protein (CRP), White Blood Cell (WBC) count, Polymorphonuclear (PNL) percentage], synovial fluid or tissue cultures, implant sonication and nuclear imaging. Even then, the diagnosis may seem likely but cannot be confirmed with certainty, leading to a continuous decision-making struggle. In addition, the use of some specific tests and biomarkers (e.g. synovial CRP, synovial alpha-defensin, implant sonication) in some institutes is limited due to their high cost or unavailability [10, 11].

The importance of novel synovial biomarkers in identifying PJIs has grown critical, particularly in situations when serum biomarkers and imaging tests are ambiguous. Synovial CRP, D-lactate, leucocyte esterase, and alpha-defensin have all been studied as preoperative or intraoperative PJI identification methods, with the latest being a reliable test with a sensitivity range of 96–100% and a specificity exceeding 90% [12]. Nevertheless, the data is controversial on whether the test provides additional evidence for PJI diagnosis when corresponding synovial fluid analysis is performed. Yet, despite its place as a standalone test in the ICM criteria of 2018, its high cost-effectiveness continues to be the key hurdle to widespread use [13].

Synovial calprotectin testing has demonstrated promising results in diagnosing PJI, even when preoperative cultures are negative. According to the most recent meta-analysis, it has a pooled specificity and sensitivity of 93% and 92%, respectively [14]. With a sensitivity of 98.1% and specificity of 95.7% at a threshold of ≥ 50 mg/L, the calprotectin point-of-care (POC) test has demonstrated exceptional performance [15]. Interestingly, investigations have shown that calprotectin performs as well as, if not better than, other recognized synovial biomarkers. Calprotectin outperformed leukocyte esterase and alpha-defensin assays in terms of sensitivity and specificity. However, its performance may vary based on the PJI defining criteria applied [16, 17].

Calprotectin concentrations in the synovial fluid can be measured using the two available methods. The calprotectin lateral flow test (LFT) detects synovial calprotectin quantitatively and has the advantage of immediate results, making it useful for intraoperative decision-making, whereas ELISA Immunoassay uses monoclonal and polyclonal antibodies against calprotectin to detect it colorimetrically. Both tests demonstrate great and comparable diagnostic accuracy, although synovial calprotectin analysis using ELISA yields statistically significantly better diagnostic indices for PJI [14]. Even so, the low cost, the wide availability, and the rapid intraoperative results make the calprotectin LFT a valuable and accurate addition to the preoperative diagnostic workup before a PJI-related surgery, especially in cases where the gold standard results are inconclusive.

Synovial calprotectin has a great diagnostic accuracy, according to four recent meta-analyses that examined the two methods of this marker for the diagnosis of a PJI [14, 18–20]. Subsequent published research has assessed the LFT accuracy. This meta-analysis aims to assess the validity and reliability of intraoperative synovial calprotectin LFT as a decision-making tool in PJI procedures.

## Materials and methods

This systematic review and meta-analysis were conducted according to the Preferred Reporting Items for Systematic Reviews and Meta-Analyses (PRISMA) guidelines [21]. The protocol was registered in the International Prospective Register of Systematic Reviews (PROSPERO) under the registration number CRD420251090158. The main target was studies that compared intraoperative synovial fluid calprotectin LFT, as a biomarker to detect PJI, with widely accepted criteria like the MSIS and ICM-2018.

### Identifying the research questions

(i) What is the role of intraoperative synovial fluid calprotectin LFT as a biomarker for decision-making in PJI-related operations? (ii) Is its validity and reliability sufficient in regard to sensitivity, specificity, diagnostic odds ratio (DOR), positive predictive value (PPV), negative predictive value (NPV), Positive/ Negative Likelihood Ratio (LR+/LR-), and area under the curve (AUC)? (iii) Could it take its place as a standalone test in the following PJI algorithms?

### Literature search

**Fig. 1:**
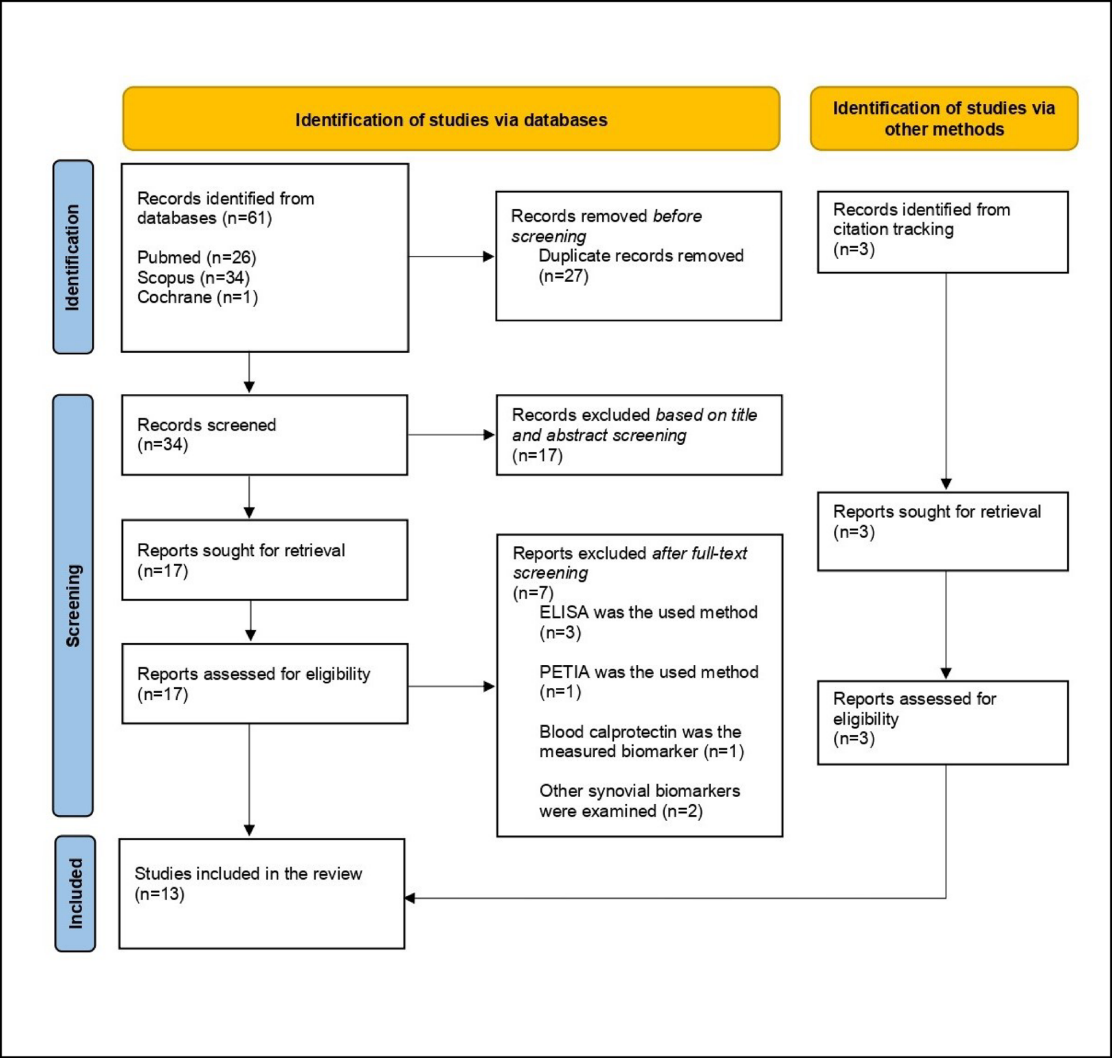
PRISMA-ScR (Preferred Reporting Items for Systematic reviews and Meta-Analyses extension for Scoping Reviews) flow diagram of the selection of studies in the review. *ELISA* Enzyme-linked Immunosorbent Assay,* PETIA* Particle Enhanced Turbidimetric Immunoassay

Electronic databases of PubMed, Elsevier Scopus and Cochrane were researched from inception to May 2025. The research algorithm included the following terms: “synovial” AND “calprotectin” AND “periprosthetic joint infection”. The articles were screened by two reviewers according to their title and abstract relevance, and full-text review was done in the articles that met the inclusion/ exclusion criteria. Supplementary studies were recognized via citation and reference lists tracking (Fig. 1).

### Study selection

#### Inclusion criteria

- Patients who underwent a possible PJI-related arthroplasty procedure (i.e. 1 or 2 stage TKR or THR/ hemiarthroplasty revision, spacer exchange or 2nd stage revision).
- Intraoperative synovial calprotectin LFT was used as a biomarker to diagnose PJI.
- Traditional standardized diagnostic criteria, such as MSIS and ICM-2018, were used for PJI detection.
- A comparison between synovial calprotectin LFT result and the diagnostic criteria was performed.
- All articles included had to be written in English.

#### Exclusion criteria

- Cases in which other methods of calprotectin measurement tests (e.g. ELISA Immunoassay) were used, instead of the LFT.
- Studies that performed the calprotectin test preoperatively, in the synovial fluid extracted through joint aspiration.
- Studies that did not use standardized diagnostic criteria were excluded.

### Study assessment and charting of the data

**Fig. 2:**
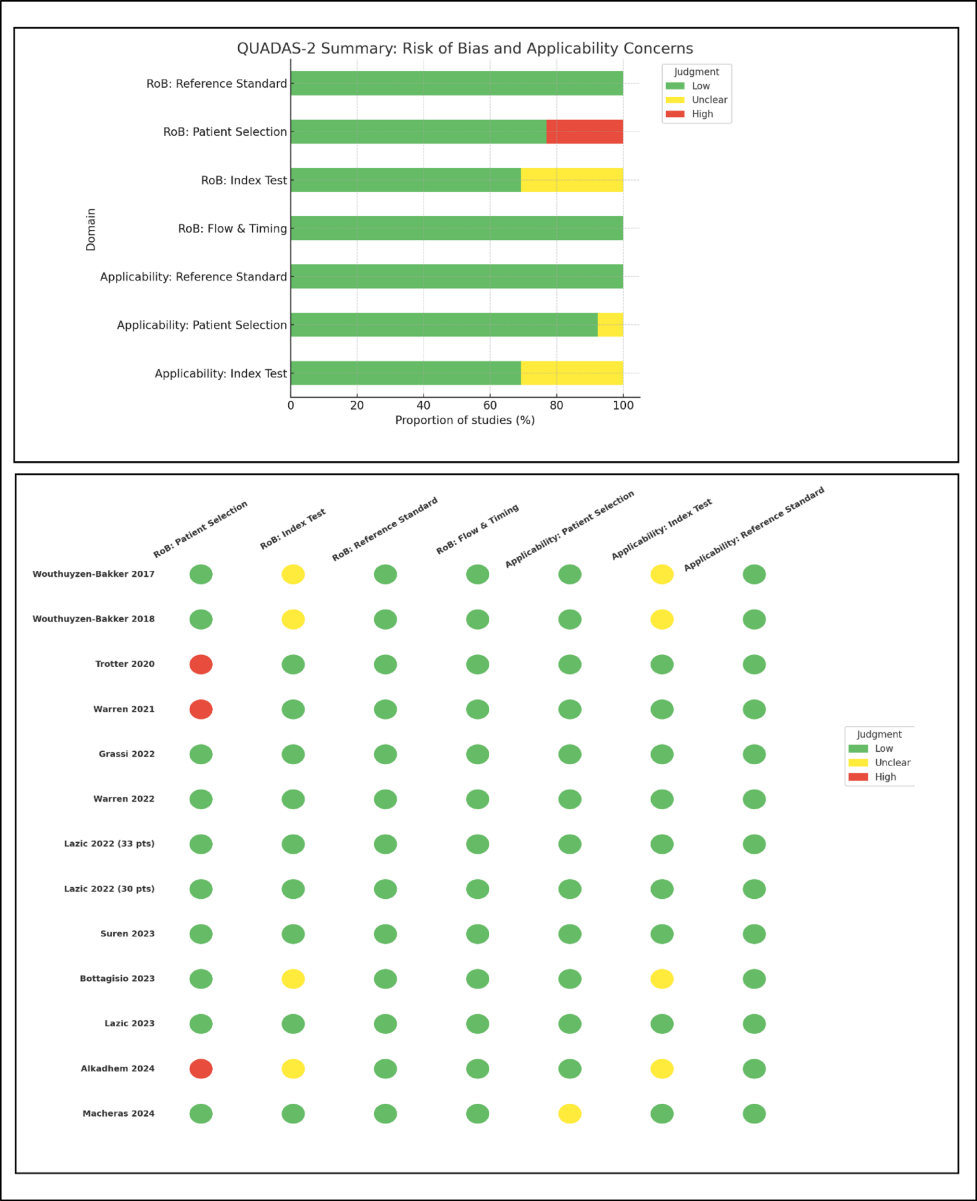
QUADAS-2 scores and traffic light table analysis for studies included in the meta-analysis

Thirteen studies were finally included in the review after completion of the full-text review phase. The relevant data (authors, year of publication, location of hospital or institute, population’s characteristics, LFT kit used, reference standard criteria used, cutoff point, mean calprotectin concentration, sensitivity, specificity, AUC, LR+, LR-, PPV, NPV, DOR) were extracted and illustrated in a data table, as displayed in Table 1.. Quality assessment of each study was performed using the QUADAS (Quality Assessment of Diagnostic Accuracy Studies) tool [22]. The QUADAS score consists of four parts: (i) patient selection, (ii) index test, (iii) reference standard, (iv) flow and timing. The risk of bias assessment and the clinical applicability of the above domains were assessed with signaling questions, answered by the first author (Fig. 2).

**Table 1:** Characteristics of the studies included in the meta-analysis

Study	Study design	Patients (Infected/ Total)according to the reference standard criteria	Age	LFT kit	Reference standard	Site (Hip/Knee/Shoulder/Elbow)	Cutoff	Sensitivity	Specificity	AUC	LR+	LR-	PPV	NPV	DOR (IC 95%)	Mean calprotectin concentration in septic VS aseptic joints
Wouthuyzen-Bakker et al. (2017) [23] Netherlands	Prospective	19/61 (31%)	Septic 65 (24–87) Aseptic 60 (23–90)	Quantum Blue® fCAL stool test kit [BÜHLMANN, Laboratories AG, Basel, Switzerland]	MSIS 2011	45/10/5/1	> = 50 mg/L	0.89	0.9	0.94	8.9	0.1	0.81	0.95	80.75(13.47-484.21)	991mg/L vs 11mg/L
Wouthuyzen-Bakker et al. (2018) [24] Netherlands	Prospective	15/52 (29%)	N/A	Quantum Blue® fCAL stool test kit [BÜHLMANN, Laboratories AG, Basel, Switzerland]	MSIS 2011	32/17/3/0	> = 50 mg/L	0.867	0.917	0.94	10.9	0.14	0.813	0.944	73.66(11.02-492.51)	859 mg/L vs 7 mg/L
Trotter et al. (2020) [25] UK	Retrospective	24/69 (35%)	74.3 (45-89)	Lyfstone synovial test kit [Lyfstone AS, Oslo, Norway]	ICM 2018	52/17/0/0	> = 14 mg/L	0.75	0.7556	0.78	3.07	0.33	0.6207	0.85	9.27 (2.94-29.20)	N/A
Warren et al. (2021) [15] USA	Retrospective	53/123 (43%)	Septic 66.9 ± 10.6 Aseptic 65.4 ± 10.6	Lyfstone synovial test kit [Lyfstone AS, Oslo, Norway]	MSIS 2013	0/123/0/0	> = 50 mg/L	0.981	0.957	0.969	22.89	0.018	0.945	0.985	1161.33 (117.37-11491.26	N/A
Grassi et al. (2022) [16] Italy	Prospective	39/89 (44%)	77	CalFast-NeXT stool test kit [Eurospital, Trieste, Italy]	ICM 2018	0/89/0/0	> = 50 mg/L	0.974	0.94	0.957	16.239	0.027	0.927	0.979	595.33 (59.49-5957.30)	N/A
Warren et al. (2022) [26] USA	Prospective	55/123 (45%)^a^	Septic 68.8 ± 10.2 Aseptic 65.1 ± 10.6	Lyfstone synovial test kit [Lyfstone AS, Oslo, Norway]	MSIS 2013ICM 2018Proposed EBJIS 2019	0/123/0/0	> = 50 mg/L	0.982	0.985	0.984	66.8	0.02	0.982	0.985	3588.00 (219.25-58716.75)	N/A
Lazic et al. (2022) [27] Germany	Prospective	17/33 (51%)	65.1 ± 13.9	Lyfstone synovial test kit [Lyfstone AS, Oslo, Norway]	EBJIS 2021	21/12/0/0	70.5 mg/ dl	0.88	0.88%	0.89	7.06	0.13	0.8824	0.875	52.50 (6.4974–424.86)	N/A
Lazic et al. (2022) [28] Germany	Prospective	14/30 (46%)	72.3 ± 14.8	Lyfstone synovial test kit [Lyfstone AS, Oslo, Norway]	EBJIS 2021	23/7/0/0	76 mg/dl	0.77	0.81	0.77	3.81	0.35	0.77	0.76	10.83 (1.96-59.84)	N/A
Suren et al. (2023) [29] Germany	Prospective	34/137 (25%)	Septic 70 ± 11Aseptic 67 ± 13	Lyfstone synovial test kit [Lyfstone AS, Oslo, Norway]	ICM 2018	53/84/0/0	85.5 mg/L	0.92	0.95	0.94	23.48	0.09	0.8857	0.9706	255.75 (54.26-1205.41)	N/A
Bottagisio et al. (2023) [30] Italy	Prospective	12/55 (22%)	Septic 70 ± 15Aseptic 72 ± 10	Quantum Blue® fCAL stool test kit [BÜHLMANN, Laboratories AG, Basel, Switzerland]	MSIS 2013ICM 2018	N/A	529.5 µg/g	0.80	0.944	0.852	11.7	0.179	N/A	N/A	40.00 (6.91-231.60)	874 µg/g vs 30 µg/g
Lazic et al. (2023) [31] Germany	Prospective	10/33 (30%)	73.3 ± 11.4	Lyfstone synovial test kit [Lyfstone AS, Oslo, Norway]	EBJIS 2021	15/18/0/0	70 mg/dl	0.60	0.78	0.71	2.76	0.51	0.5455	0.8182	5.40 (1.08-26.93)	N/A
Alkadhem et al. (2024) [32] The Netherlands	Retrospective	19/137 (14%)^b^	71 (42-92)	Quantum Blue® fCAL stool test kit [BÜHLMANN, Laboratories AG, Basel, Switzerland]	EBJIS 2021	62/75/0/0	> = 50 mg/L	1,00	0.79	0.96	4.7	0,00	0.43	1,00	143.10 (8.35- 2450.16)	N/A
Macheras et al. (2024) [33] Greece	Prospective	26/48 (54.1%)	65.8 (51-79)	Lyfstone synovial test kit [Lyfstone AS, Oslo, Norway]	EBJIS 2021	48/0/0/0	> = 50 mg/L	0.962	0.909	0.917	10.6	0.04	0.926	0.952	250.00 (21.12- 2959.81)	119.9 mg/L vs 31.6 mg/L
Pooled		337/990 (34%)						0.88 (0.82-0.92)	0.89 (0.83-0.93)	0.94 (0.91-0.96)	7.9 (5.2-12.0)	0.14 (0.09-0.21)	0.83 (0.76-0.88)	0.93 (0.89-0.96)	56.10 (28.40-110.80)	

*LFT* Lateral Flow Test, *DOR* Diagnostic Odds Ratio,*PPV* Positive Predictive Value, *NPV* Negative Predictive Value, *LR+* Positive Likelihood Ratio, *LR-* Negative Likelihood Ratio, AUC; Area Under the Curve, MSIS; Musculoskeletal Infection Society, *ICM* International Consensus Meeting, *EBJIS* European Bone and Joint Infection Society

^a^Three system criteria were analyzed, but only the ICM 2018 that had the best performance was included in the analysis

^b^Calprotectin LFT was used in 88 out of 137 cases, while the remaining 49 were examined using a calprotectin ELISA kit. Nevertheless, the authors included both test results in their analysis

### Data analysis

Statistical analyses were conducted using Python (v3.11) with the *statsmodels* package. A random-effects meta-analysis was performed using the DerSimonian-Laird method. Pooled estimates for sensitivity, specificity, LR+, LR-, DOR and AUC were calculated with 95% confidence intervals (CI). The diagnostic accuracy of calprotectin was assessed by AUC. Although bivariate and Hierarchical Summary Receiver Operating Characteristic (HSROC) models are commonly used for diagnostic meta-analyses, a univariate random-effects approach was adopted here. This method provided a robust estimation of pooled metrics, while allowing for a detailed assessment of heterogeneity by calculating the inconsistency index (*I*^2^) for the DOR. Subgroup analyses compared performance by test kit type (Lyfstone vs. Quantum Blue^®^) and cutoff threshold (≥ 50 mg/L vs. other values).

### Ethical consideration

As this study involved the synthesis of data from previously published research and did not include any new data collection involving human participants, it was deemed exempt from institutional review board (IRB) approval.

## Results

A total of 61 articles (26 from PubMed, 34 from Scopus, and 1 from Cochrane Library) were identified from the research algorithm. After 27 duplicate articles were excluded, the remaining 34 studies were screened using the inclusion and exclusion criteria by reviewing the title and abstract. The remaining 17 articles were qualified for full-text retrieval and review. This resulted in 10 studies that met al.l of the criteria. Hand searching through citation tracking identified a further 3 new relevant articles. The final number of the studies included in the meta-analysis was 13 [15, 16, 23–33] (Fig. 1).

The 13 studies consisted of 990 patients who had undergone hip, knee, shoulder, or elbow joint replacement, including 337 patients with PJI and 653 patients with non-PJI according to the reference standard criteria. The studies were published between January 2017 and May 2024. Eight studies used a synovial calprotectin LFT kit (*Lystone® Calprotectin*,* Lyfstone AS*,* Oslo*,* Norway*), while the other 5 studies used a stool calprotectin LFT kit (*Quantum Blue*^®^,* BÜHLMANN*,* Laboratories AG*,* Basel*,* Switzerland* or *CalFast-NeXT*,* Eurospital*,* Trieste*,* Italy*) for the detection and measurement of synovial calprotectin. It should be noted that the Quantum Blue^®^ calprotectin assay was originally developed and validated for fecal calprotectin measurement, and its application to synovial fluid represents an off-label use, as reported in the included studies [16, 23, 24, 30, 32]. Eleven studies were conducted prospectively and 2 retrospectively. Three studies applied the Musculoskeletal Infection Society (MSIS) as the diagnosis standard, 5 applied the European Bone and Joint Infection Society (EBJIS), 3 applied the International Consensus on Infection (ICM), one applied both MSIS and ICM, and one applied all three criteria. Detailed characteristics and the results of each study are shown in Table 1.. The results of the QUADAS-2 are in Fig. 2.

**Fig. 3:**
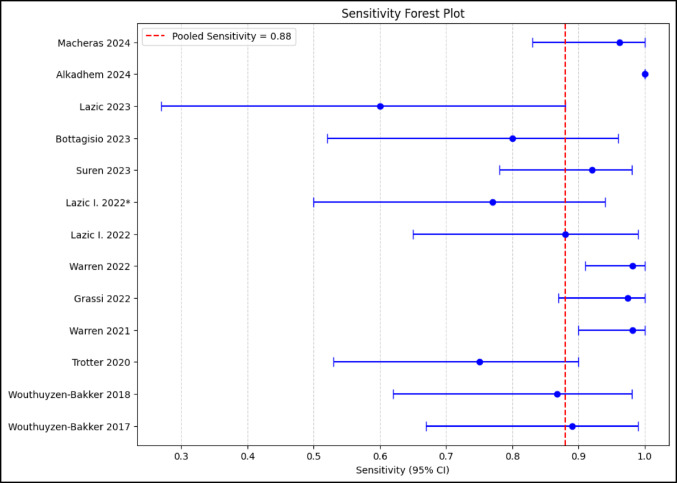
Pooled sensitivity of calprotectin LFT for PJI

**Fig. 4:**
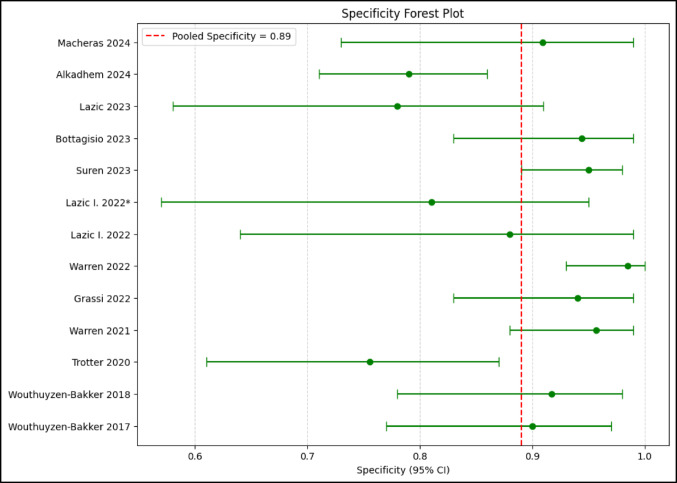
Pooled specificity of calprotectin LFT for PJI

**Fig. 5:**
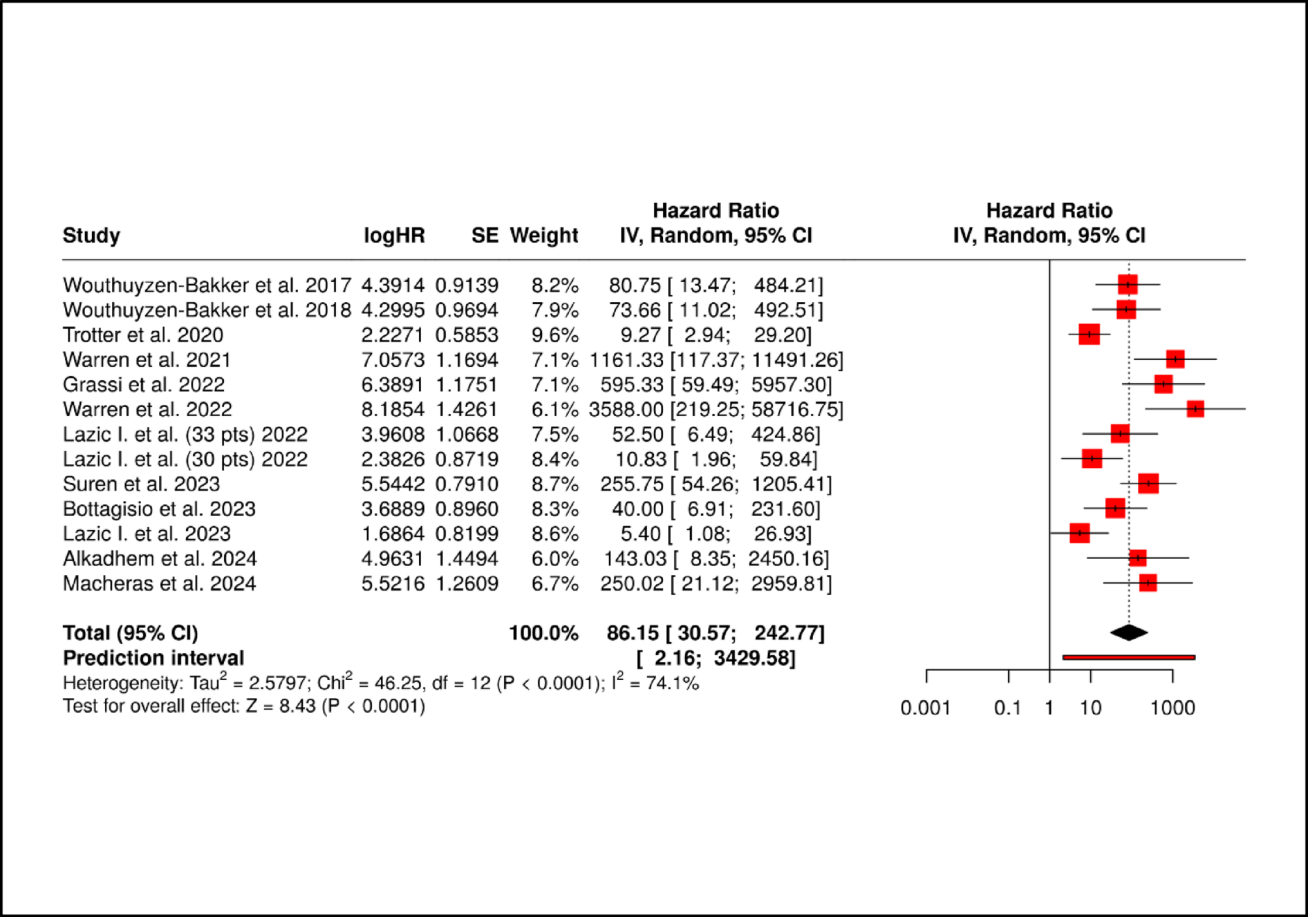
Forest plot showing the study-specific and meta-analyzed estimates for risk ratio utilizing the Fixed-effect model

**Fig. 6:**
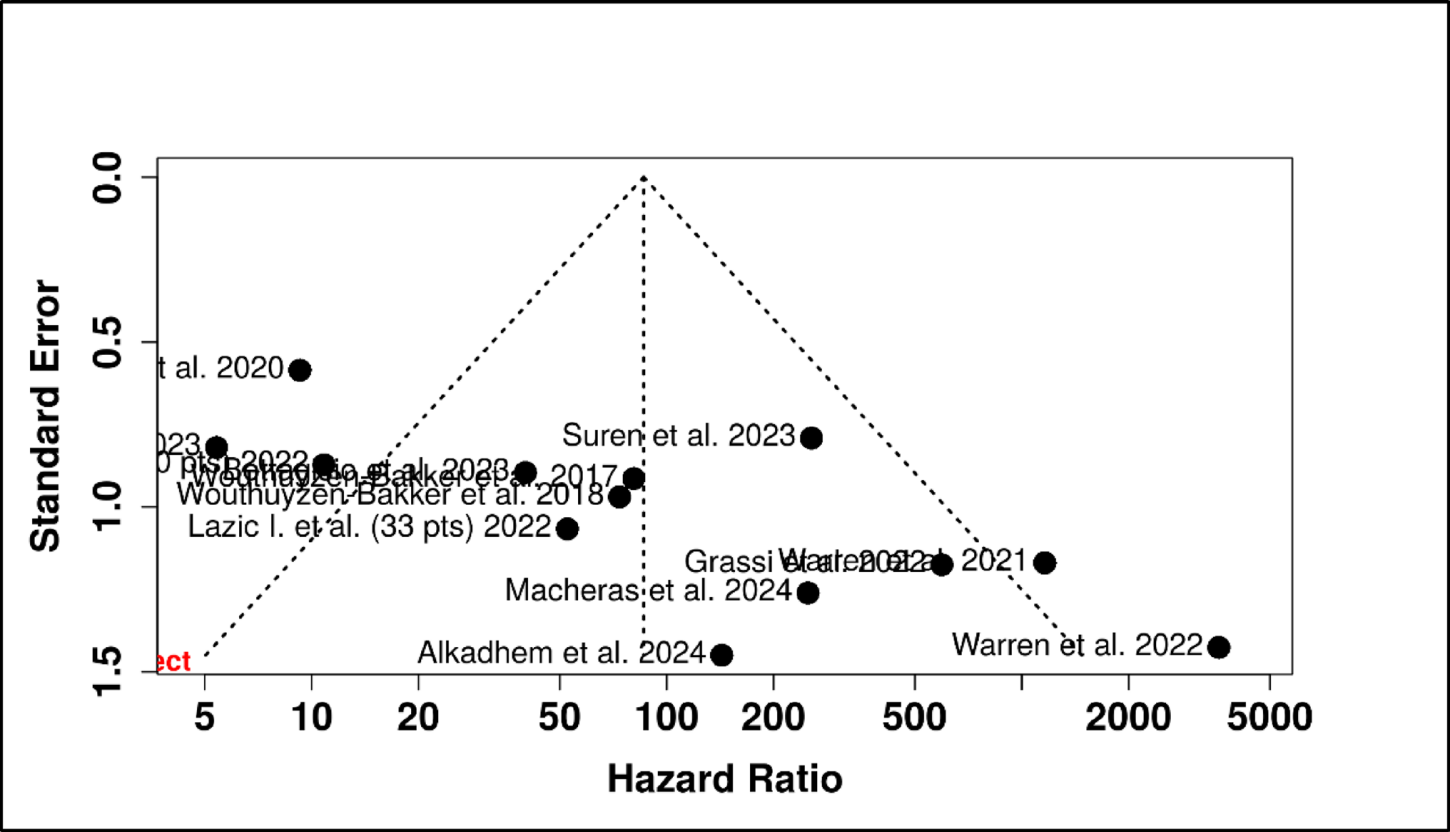
Funnel plot assessing publication bias in the meta-analysis

The pooled sensitivity, specificity, PPV, NPV, LR+, LR-, and DOR were 0.88 (95% CI 0.82–0.92), 0.89 (95% CI 0.83–0.93), 0.83 (95% CI 0.76–0.88), 0.93 (95% CI 0.89–0.96), 7.9 (95% CI 5.2–12.0), 0.14 (95% CI 0.09–0.21), and 56.1 (95% CI 28.4–110.8.4.8), respectively (Figs. 3 and 4). The AUC was 0.94 (95% CI 0.91–0.96). The corresponding I^2^ statistics for the DOR (*Tau²* = 2.58, *p* < 0.001) was 74.1% (95% CI 12.1–91.0%) indicating that there was substantial heterogeneity between studies (Figs. 5 and 6). We performed subgroup analysis according to test kit type (Lyfstone vs. Quantum Blue^®^) and cutoff threshold (≥ 50 mg/L vs. other values) to ascertain the potential source of heterogeneity. Quantum Blue^®^ group demonstrated marginally higher sensitivity (91 vs. 86%), whereas Lyfstone group showed slightly better specificity (90 vs. 88%). No significant difference was observed for all comparisons (*p* > 0.05). All subgroups maintained AUC > 0.90, confirming robust diagnostic utility. The ≥ 50 mg/L cutoff subgroup demonstrated significantly superior diagnostic performance across all metrics compared to other thresholds (*p* ≤ 0.005). I² remained high (68–75%) across subgroups, suggesting residual variability from unmeasured factors (e.g. mixed reference standards, joint type). Results of the subgroup analysis are presented in Table 2.

**Table 2:** Subgroup analysis of calprotectin for PJI diagnosis

Subgroup	Studies (*n*)	Sensitivity (95% CI)	Specificity (95% CI)	LR+ (95% CI)	LR- (95% CI)	AUC (95% CI)	DOR (95% CI)	I² (DOR)
All studies	13	0.88 (0.82–0.92)	0.89 (0.83–0.93)	7.9 (5.2–12.0)	0.14 (0.09–0.21)	0.94 (0.91–0.96)	56.1 (28.4–110.8)	74.1%
*By LFT Kit*								
Quantum Blue^®^	4	0.91 (0.82–0.96)	0.88 (0.76–0.95)	8.1 (4.3–15.4)	0.10 (0.05–0.21)	0.95 (0.91–0.98)	73.3 (22.1–243.2)	68.0%
Lyfstone	8	0.86 (0.78–0.92)	0.90 (0.84–0.94)	7.8 (4.8–12.6)	0.16 (0.10–0.25)	0.93 (0.89–0.96)	48.5 (22.4–105.1)	75.0%
*By Cutoff*								
≥ 50 mg/L	7	0.90 (0.84–0.94)	0.91 (0.85–0.95)	9.8 (6.1–15.8)	0.11 (0.07–0.18)	0.95 (0.92–0.97)	68.9 (31.2–152.1)	70.3%
Other cutoffs	6	0.83 (0.72–0.90)	0.85 (0.76–0.91)	5.4 (3.3–8.8)	0.20 (0.12–0.33)	0.91 (0.86–0.94)	32.4 (11.2–93.7)	74.8%

*DOR* Diagnostic Odds Ratio,* LR+* Positive Likelihood Ratio,* LR-* Negative Likelihood Ratio,* AUC* Area Under the Curve,* I*^2^ I-squared statistic

## Discussion

This systematic review and meta-analysis demonstrates the high accuracy and reliability of synovial calprotectin LFT as a tool for the intraoperative assessment of PJI. With pooled sensitivity and specificity of 88% and 89%, respectively, and an AUC of 0.94, our findings confirm the strong diagnostic potential of calprotectin LFT, especially when standard diagnostic methods yield inconclusive results.

The pooled PPV and NPV were calculated at 0.83 and 0.93, respectively. It should be noted that while sensitivity and specificity are intrinsic properties of the calprotectin LFT, PPV and NPV are inherently dependent on the prevalence of PJI within the studied population. Consequently, in clinical settings with a high pre-test probability of infection, the PPV is expected to increase, whereas in low-prevalence environments, the NPV will be further optimized, reinforcing the test’s utility as a reliable tool for ruling out infection.

The diagnostic accuracy of synovial calprotectin LFT observed in our analysis aligns with the results of previous meta-analyses. Peng et al. reported comparable pooled sensitivity, specificity, and AUC values (94%, 93%, and 0.98, respectively) across both ELISA and LFT results, confirming the robustness of calprotectin as a synovial biomarker [20]. The meta-analyses of Xing et al. (94%, 93%, and 0.98, respectively) and Hantouly et al. (92%, 93%, and 0.935, respectively) concluded in similar results, as well [18, 19]. The most recent meta-analysis of Festa et al. included 14 studies with 902 patients, and resulted in 92% sensitivity, 93% specificity and an AUC of 0.93. Following the subgroup analysis, even though the ELISA group had increased pool sensitivity, specificity and AUC compared to the LFT group (96%, 97%, and 0.968 vs. 90%, 92%, and 0.915, respectively), no statistical differences were found in terms of sensitivity and specificity, between the two groups [14]. In addition, while ELISA has achieved marginally better performance in some studies, the rapid turnaround, lower cost, and intraoperative applicability of LFT make it more suitable for real-time clinical decision-making in PJI-related arthroplasty operations [16, 34, 35]. This is the first meta-analysis that exclusively assesses the reliability of intraoperative synovial calprotectin LFT for PJI detection, in terms of sensitivity, specificity, PPV, NPV, LR+, LR-, and AUC.

In our subgroup analysis, we compared the accuracy of the two most commonly used calprotectin LFT kits, Lyfstone and Quantum Blue^®^. The Quantum Blue^®^ group demonstrated increased pooled sensitivity compared to the Lyfstone group (91 vs. 86%), while Lyfstone showed slightly higher specificity (90 vs. 88%). Both kits achieved high AUC values exceeding 0.90, confirming their diagnostic utility in identifying PJI. Although no statistically significant difference was observed between the groups (*p* > 0.05), these small variations in diagnostic values may reflect differences in kit calibration, or cutoff interpretation. More importantly, both kits performed excellently, reinforcing the reliability of calprotectin LFT and its widespread use.

The diagnostic performance of synovial calprotectin LFT was statistically significantly influenced by the threshold applied in the included studies. Articles using a cutoff of ≥ 50 mg/L had superior pooled sensitivity (90 vs. 83%), specificity (91 vs. 85%), and AUC (0.95 vs. 0.91) compared to studies applying lower or different thresholds. These findings highlight the importance of threshold standardization, as inconsistent cutoffs may contribute to inter-study heterogeneity. Our meta-analysis results suggest that using a cutoff of ≥ 50 mg/L is the best choice for balancing sensitivity and specificity when diagnosing PJI based on LFT during surgery.

There are some limitations in the meta-analysis. First, the number of articles included, and their sample size is limited. Second, despite the overall high diagnostic accuracy observed, our analysis revealed substantial heterogeneity among the included studies, as indicated by the I² value of 74.1% for the DOR. While subgroup analyses attempted to elucidate the sources of variability, examining factors such as test kit type and cutoff thresholds, heterogeneity remained consistently high across all subgroups (I² 68–75%). This residual heterogeneity could not be fully explained by the parameters evaluated and likely reflects the influence of unmeasured variables, such as differences in patient populations (e.g., joint type, infection chronicity, comorbidities), perioperative protocols, or interpretation of the reference standard definitions (MSIS, ICM, EBJIS). An additional limitation is that several included studies used calprotectin LFT assays originally developed and validated for fecal samples, and their application to synovial fluid represents an off-label use, which may introduce variability related to matrix effects and assay calibration. Furthermore, the inclusion of both prospective and retrospective studies, and possible differences in sample handling or synovial fluid quality, may have contributed to inconsistency. These findings underscore the necessity of future multicenter trials with standardized diagnostic pathways and uniform application of the LFT methodology to mitigate the impact of such variability.

In clinical practice, the synovial calprotectin LFT offers clear advantages in accessibility, requiring minimal operator training and yielding results within 15 min without the need for laboratory infrastructure [15, 33]. Its relatively low cost compared to alpha-defensin testing makes it particularly suitable for routine use in both high- and low-resource surgical settings. Reported costs for synovial calprotectin LFTs typically range between €20 and €50 per test, whereas alpha-defensin LFTs generally cost between €80 and €400. It is imperative to note, however, that these cost estimates are purely indicative and remain subject to substantial fluctuation contingent upon geographic location, localized market dynamics, divergent healthcare reimbursement frameworks, and institutional procurement agreements [10, 13, 14, 24, 36].

## Conclusion

Intraoperative synovial calprotectin LFT shows a high level of diagnostic accuracy for PJI detection. With pooled sensitivity and specificity values approaching 90% and an AUC of 0.94, calprotectin LFT emerges as a valuable point-of-care tool for real-time decision-making in arthroplasty procedures. Subgroup analysis supports the use of a cutoff value of ≥ 50 mg/L and indicates consistent performance across different commercial kits. However, significant heterogeneity was observed across studies, suggesting that unmeasured factors may still have an unpredictable impact on diagnostic accuracy. This highlights the need for further standardization in future relevant research, even though current evidence strongly supports the incorporation of calprotectin LFT into PJI diagnostic algorithms as a supportive adjunctive tool, particularly for assisting in clinical decision-making, when the gold-standard results are inconclusive.

## Data Availability

No datasets were generated or analysed during the current study.
